# Sphenoid Brown Tumor Associated with a Parathyroid Carcinoma

**DOI:** 10.1155/2014/837204

**Published:** 2014-09-08

**Authors:** Abelardo Loya-Solís, Alejandra Mendoza-García, Luis Ceceñas-Falcón, René Rodríguez-Gutiérrez

**Affiliations:** ^1^Pathology Department, University Hospital “Dr. José E. González” and Medical School of the Universidad Autónoma de Nuevo León, Avenida Madero y Avenida Gonzalitos s/n, Colonia Mitras Centro, 64460 Monterrey, NL, Mexico; ^2^Internal Medicine Department, University Hospital “Dr. José E. González” and Medical School of the Universidad Autónoma de Nuevo León, Avenida Madero y Avenida Gonzalitos s/n, Colonia Mitras Centro, 64460 Monterrey, NL, Mexico; ^3^Endocrinology Division, University Hospital “Dr. José E. González” and Medical School of the Universidad Autónoma de Nuevo León, Avenida Madero y Avenida Gonzalitos s/n, Colonia Mitras Centro, 64460 Monterrey, NL, Mexico

## Abstract

Brown tumors are osteolytic bone lesions that occur as a result of persistent hyperparathyroidism. They usually appear late in the natural history of the disease and are currently very rare due to an earlier diagnosis of primary hyperparathyroidism. We present the case of a 53-year-old female with a 2-month history of bitemporal hemianopsia and diplopia. A computed tomography showed an osteolytic bone lesion that involved the sphenoid corpus and clivus. A biopsy was made and the histopathology result was consistent with a brown tumor. The aforementioned location is very infrequent to such tumors, and therefore represented a diagnostic challenge. However, in this case, its association to primary hyperparathyroidism was the clue for the diagnosis. The association of a brown tumor secondary to a parathyroid carcinoma has been seldom reported. This case represents, to our knowledge, one of the few brown tumors described in such location and the first secondary to a parathyroid carcinoma.

## 1. Introduction

Primary hyperparathyroidism is the most common cause of hypercalcemia in the ambulatory setting [1]. Since the introduction of serum calcium into routine laboratory tests its clinical presentation has changed from being defined by its classic signs and symptoms to, nowadays, often discovered accidentally while the patient is still asymptomatic [2]. Parathyroid adenoma is the most common etiology (85%), followed by parathyroid hyperplasia (14%), and parathyroid carcinoma represents ≤1% of the cases [3]. Unlike adenomas, parathyroid carcinomas present equally between the two genders and are associated with higher calcium levels (≥14.0 mg/dL), neck mass (35%), bone disease (50%), renal disease (30%), and pancreatitis (15%) [4, 5].

Osteitis fibrosa cystica is the most characteristic manifestation of hyperparathyroidism in the bone. It is characterized by the presence of intense subperiosteal resorption in the digits, skull, and long bones along with diffuse osteopenia [5]. Brown tumors represent a localized manifestation of osteitis fibrosa cystica and the most common affected sites are mandible, maxilla, clavicle, ribs, and pelvic bones. They have been reported to occur in 4.5% of patients with primary hyperparathyroidism, usually secondary to a parathyroid adenoma or hyperplasia [6]. Histologically, brown tumors are composed of multiple multinucleated giant cells arranged diffusely or in groups mixed with mononuclear stromal cells with oval to spindle morphology and hemosiderin deposition [7]. Although primary hyperparathyroidism is detected earlier, nowadays, brown tumors are an entity that might be encountered and its recognition and diagnosis are important in order to avoid unnecessary procedures and to initiate a prompt treatment.

The association of a brown tumor secondary to a parathyroid carcinoma has been seldom reported. Herein we present the case of a 53-year-old female with severe hypercalcemia and a sphenoid brown tumor secondary to a parathyroid carcinoma.

## 2. Case Report

**Figure 1:**
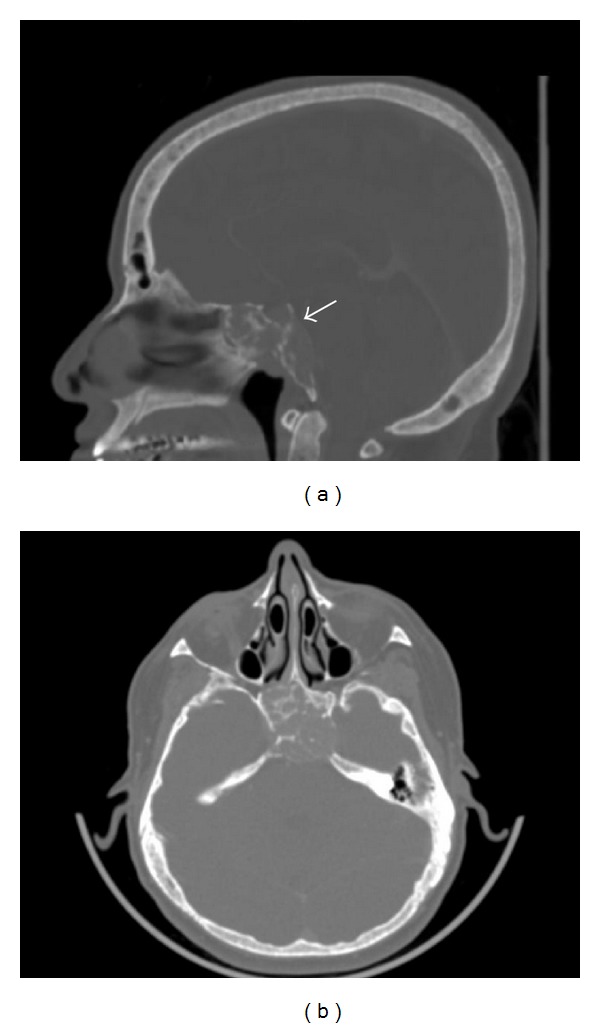
Computed tomography of skull that shows a heterogeneous, osteolytic lesion that involves sphenoid corpus and clivus. (a) Sagittal section. (b) Axial section.

A 53-year-old female consulted for assessment and management of a 2-month history of bitemporal hemianopsia and diplopia, associated with fatigue, generalized weakness, and somnolence. She had a past medical history of hypertension, nephrolithiasis, and chronic constipation. As an initial method of evaluation an enhanced computed tomography of skull was performed and it revealed a 3.0 × 3.0 × 2.0 cm, heterogeneous osteolytic lesion that involved sphenoid corpus and clivus (Figure 1).

**Figure 2:**
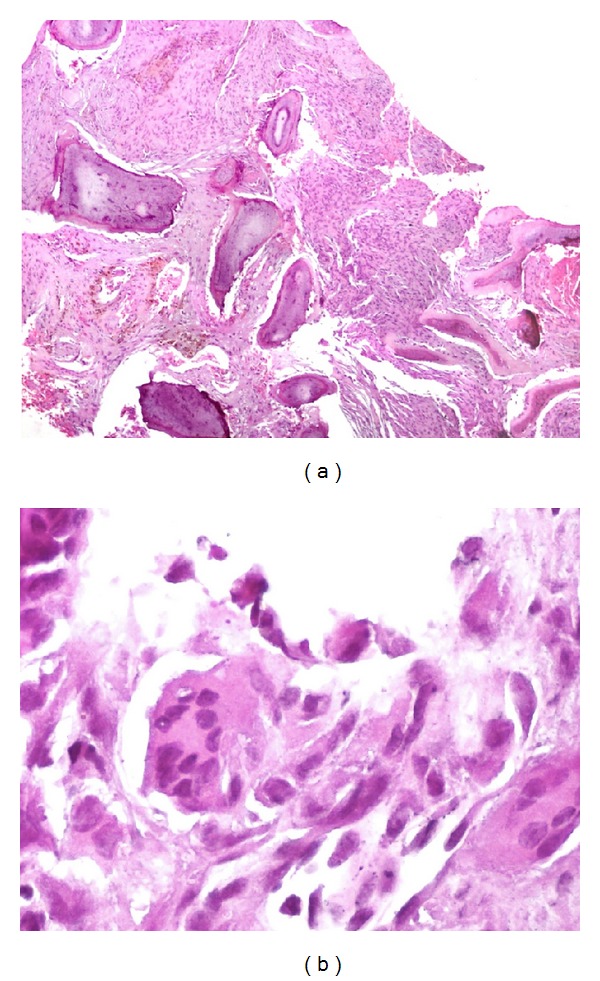
(a) Bone lesion composed of multiple multinucleated giant cells mixed with stromal spindle cells with hemosiderin deposition. H&E stain, ×50. (b) Multinucleated giant cell surrounded by stromal spindle cells. H&E stain, ×400.

**Figure 3:**
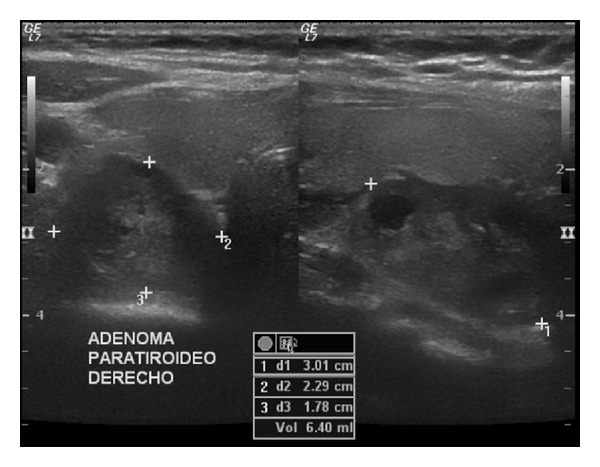
Right inferior parathyroid gland (ultrasonography).

**Figure 4:**
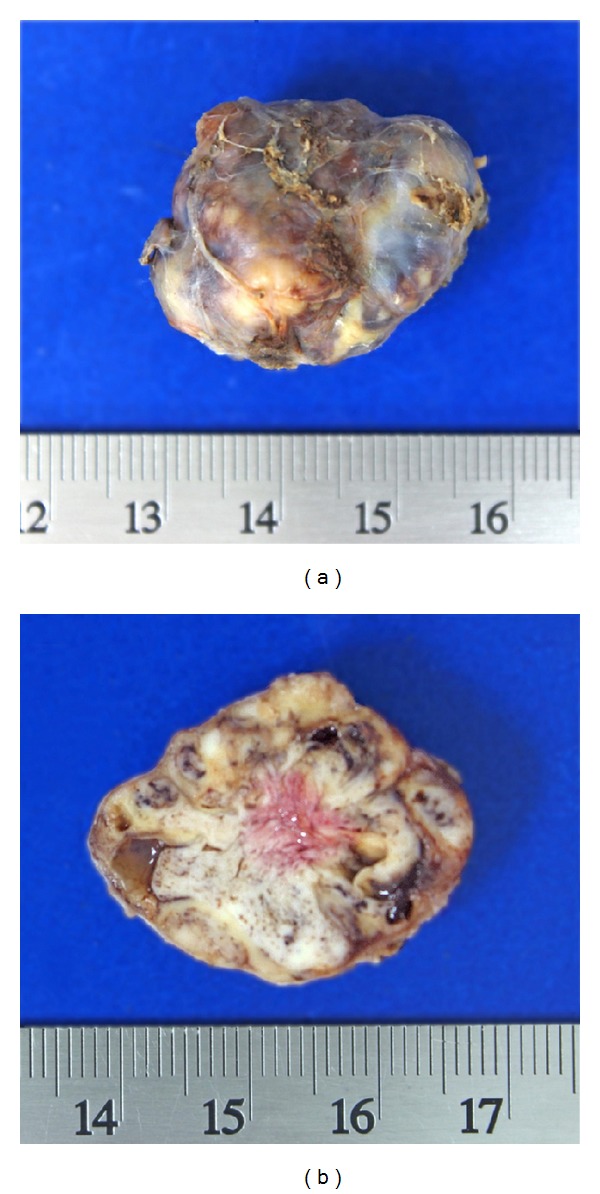
(a) Right inferior parathyroid gland. (b) Hemorrhagic and cystic areas.

**Figure 5:**
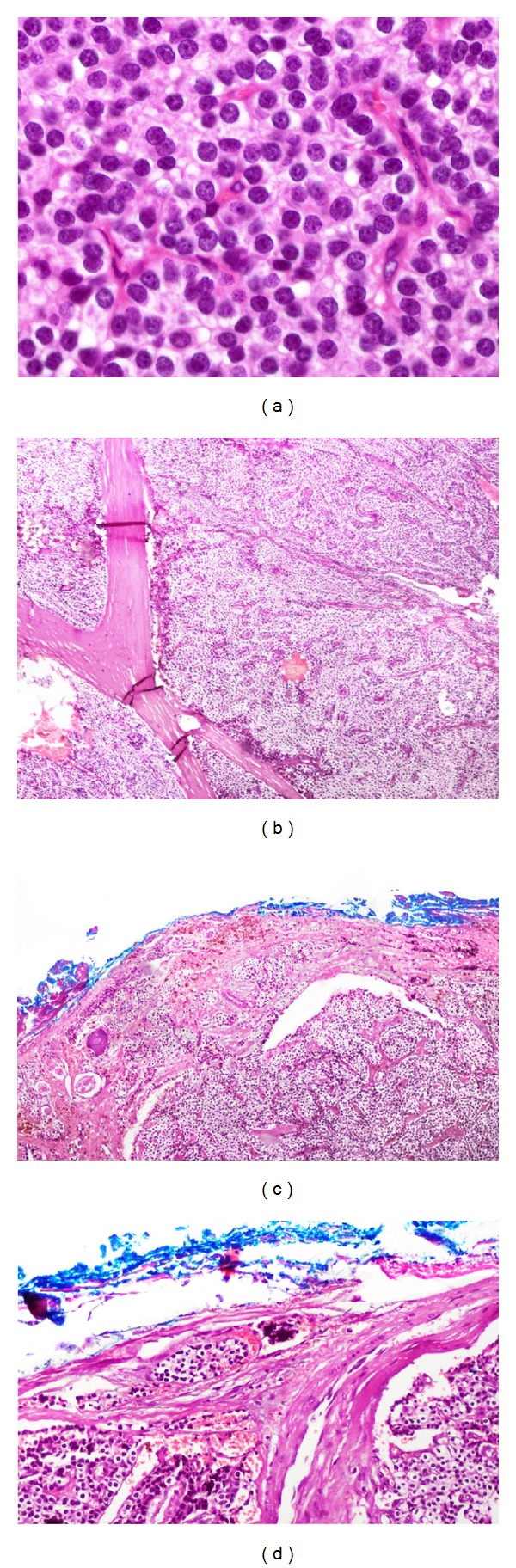
(a) Sheets of polygonal cells with clear cytoplasm and monotonous nuclei without atypia, H&E stain, ×400. (b) Thick fibrous bands, H&E stain, ×50. (c) Focal capsular invasion without perforation, H&E stain, ×50. (d) Vascular invasion, H&E stain, ×100.

Laboratory tests showed a normochromic normocytic anemia (9.67 g/dL) and serum calcium was 15.5 mg/dL (8.2–10.2), phosphorous 1.0 mg/dL (2.3–4.7), albumin 3.9 mg/dL (3.5–5.0), parathyroid hormone (PTH) 1233 pg/mL (10–55), alkaline phosphatase 262 UI/L (50–120), magnesium 1.5 mg/dL (1.5–2.5), and urinary calcium 320 mg/24 hrs (100–250). Serum levels of 25-hydroxyvitamin D and 1,25-dihydroxyvitamin D were within normal range. Glucose, creatinine, blood urea nitrogen, and calculated glomerular filtration rate were normal. See Table 1. Medical management for hypercalcemia was based on hydration, furosemide, and zoledronic acid. An endoscopic biopsy of the sphenoidal lesion was performed. Histopathological examination revealed a tumor composed of multiple multinucleated giant cells mixed with stromal spindle cells with hemosiderin deposition (Figure 2). In view of these histopathological findings a giant cell lesion was diagnosed, suggesting a brown tumor in the context of hyperparathyroidism as the most plausible cause. A neck ultrasonography was performed and it revealed an enlarged right inferior parathyroid gland (3 × 2.3 × 1.8 cm) (Figure 3). A Tc99-Sestamibi scan confirmed the hyperfunction of the same gland. In view of these findings primary hyperparathyroidism was diagnosed and the patient underwent an ipsilateral hemithyroidectomy and parathyroidectomy. Gross examination of the tumor showed a gray, oval tumor on the external surface; upon section, its consistency was firm and its cut surface was heterogeneous with white areas alternating with hemorrhagic and cystic areas (Figure 4). Microscopically the tumor was composed of sheets of polygonal cells with clear cytoplasm and monotonous nuclei with high nucleus to cytoplasm ratio and some areas showed thick fibrous bands, focal capsular invasion without perforation, and vascular invasion (Figure 5); based on these findings the diagnosis was a parathyroid carcinoma. There was no evidence of thyroid infiltration or metastases. After the surgery, PTH immediately decreased to 8.0 pg/mL and later on the patient developed a hungry bone syndrome, which was treated with calcium and vitamin D. At 6-month follow-up, visual fields improved and the patient was asymptomatic with normal serum calcium.

## 3. Discussion

In most cases, brown tumors are recognized once hyperparathyroidism has been diagnosed, although they can rarely be the initial manifestation. When brown tumors are localized in the head and neck region they are usually found in the mandible [8–10]. Only four cases have been reported in the sphenoid bone, two cases due to primary hyperparathyroidism and two cases due to secondary hyperparathyroidism [11–14]. The association of brown tumors and parathyroid carcinoma has been seldom reported due to the fact that brown tumors are formed slowly, late in the natural history of primary hyperparathyroidism [1–4]. In this sense, the rapid growth and progression of a parathyroid carcinoma rarely provide the enough time for a brown tumor to develop. This case represents, to our knowledge, one of the few brown tumors described in a parathyroid carcinoma and the first in such location. Parathyroid carcinoma constitutes < 1% of cases of primary hyperparathyroidism and it is usually associated with HPRT2 (CDC73) mutation (tumor suppressor gene). Characteristically, it has a faster progression and higher PTH and calcium levels along with a higher prevalence of renal and pancreatic disease than adenomas. Also, there is a higher incidence of bone disease that is characterized by osteopenia/osteoporosis, pathologic fractures, and rarely osteitis fibrosa cystica (brown tumors) [4, 15].

It is important to mention that the pathological diagnosis of a parathyroid carcinoma represents a diagnostic challenge even for an experienced pathologist. In the absence of metastases the two key features for its diagnosis are vascular and/or capsular invasion. Our case presented both of them and the diagnosis of parathyroid carcinoma was based on the criteria postulated by Fletcher [16].

The first-line treatment for parathyroid carcinoma is surgery (en bloc resection of the tumor). Recurrence rate at 5 and 10 years is 80–90%, respectively, with its most frequent location being at the neck [15, 17]. During follow-up close monitoring of calcium levels is warranted and, in case of recurrence, surgery is still the first therapeutic option. Radiotherapy and chemotherapy have been used in some cases with modest results [17]. In case of metastases and refractory hypercalcemia and due to the fact that hypercalcemia is the main cause of morbidity and mortality, cinacalcet, pamidronate, or denosumab can be used [18, 19]. Even though usually associated with having an ominous prognosis, survival rates may be improving with a recently reported 5-year survival rate of over 50% [4, 20]. In our case, PTH and calcium dropped immediately after surgery and at two-month follow-up calcium serum levels were within normal range.

## 4. Conclusion

The association between a parathyroid carcinoma and brown tumor has been seldom reported. We have described the case of a patient with primary hyperparathyroidism secondary to a parathyroid carcinoma with a brown tumor in the sphenoid corpus and clivus. This is a very infrequent location and it represents a diagnostic challenge. In this case, the bitemporal hemianopsia and diplopia along with the severe hypercalcemia and high PTH levels were the clues for the diagnosis. The mechanism responsible for this rapid brown tumor formation still remains to be elucidated.

## Figures and Tables

**Table 1:** Laboratory measures.

Value	Basal	After surgery	Range
Glucose (mg/dL)	78	80	(70–100)
Creatinine (mg/dL)	0.7	0.9	(0.6–1.2)
Urea nitrogen (mg/dL)	11	14	(8–23)
MDRD GFR (mL/min)	121	94	(≥60)
Albumin (g/dL)	3.9	3.8	(3.5–5.0)
Calcium (mg/dL)	15.5	9.0	(8.2–10.2)
Phosphorus (pg/mL)	1.0	3.8	(2.3–4.7)
Magnesium (mg/dL)	1.5	1.9	(1.5–2.5)
Potassium (mmol/L)	3.7	4.2	(3.5–5.0)
Urinary calcium (mg/24 h)	320	125	(100–250)
Alkaline phosphatase (UI/L)	262	140	(50–120)
PTH (pg/mL)	1233	8.0	(10–55)
25(OH)D_3_ (ng/mL)	31.0	—	(>20)
1,25(OH)_2_D_3_ (pg/mL)	37.0	—	(18–38)

MDRD GFR: modification of diet in renal disease glomerular filtration rate; PTH: parathyroid hormone; PTHrP: parathyroid hormone related protein; 25(OH)D_3_: 25,hydroxyvitamin D; 1,25(OH)_2_D_3_: 1,25-dihydroxyvitamin D.
